# Supplementary material for: Identification of X-chromosomal genes that drive sex differences in embryonic stem cells through a hierarchical CRISPR screening approach
Source: Genome Biol. 2021 Apr 16;22:110. doi: 10.1186/s13059-021-02321-2 (PMC8051100; doi:10.1186/s13059-021-02321-2)
Supplement: Supplementary file 1 — Additional file 1: Figure S1. Identification of X-chromosomal MAPK regulators through a pooled CRISPR knockout screen. Figure S2. Secondary CRISPR screens profiling pluripotency factors, differentiation kinetics and Mek phosphorylation. Figure S3. Sex differences in embryonic stem cells and mouse embryos. Figure S4. Perturbation of Klhl13 and Dusp9 in mESCs. Figure S5. Heterozygous mutations of Klhl13 and Dusp9 in female mESCs partially phenocopy the male pluripotency state. Figure S6. Identification of Klhl13 target proteins that mediate its effect on pluripotency and differentiation. Figure S7. Effects of putative Klhl13 targets proteins on MAPK target gene and pluripotency factor expression. [file 13059_2021_2321_MOESM1_ESM.pdf]

Figure S1

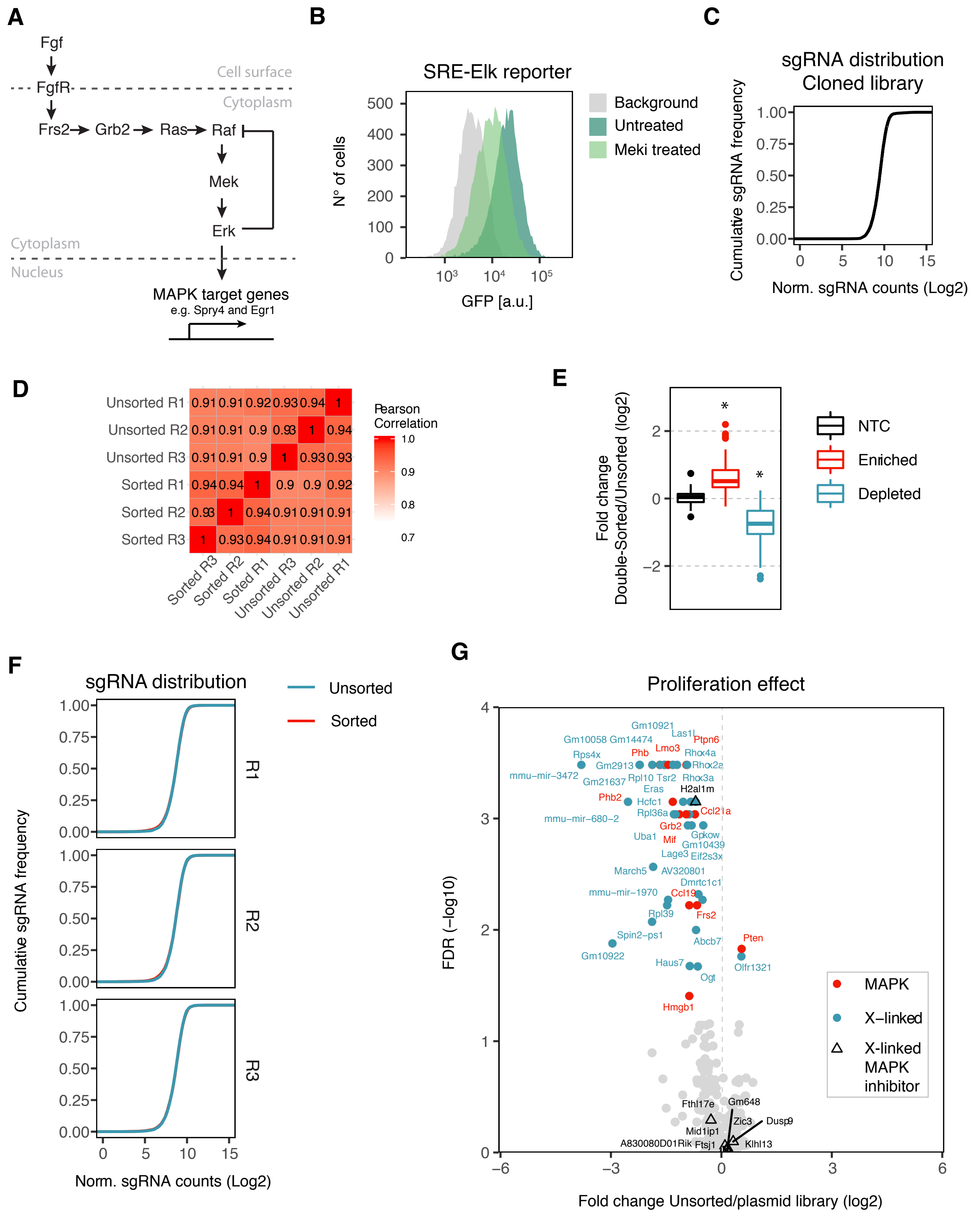

A
Fgf
FgfR
Cell surface
Cytoplasm
Frs2 Grb2 Ras Raf
Mek
Erk
Cytoplasm
Nucleus
MAPK target genes
e.g. Spry4 and Egr1
B
SRE-Elk reporter
N° of cells
GFP [a.u.]
Background
Untreated
Meki treated
C
sgRNA distribution
Cloned library
Cumulative sgRNA frequency
Norm. sgRNA counts (Log2)
D
Pearson Correlation
Unsorted R1
Unsorted R2
Unsorted R3
Sorted R1
Sorted R2
Sorted R3
E
Fold change Double-Sorted/Unsorted (log2)
NTC
Enriched
Depleted
F
sgRNA distribution
Unsorted
Sorted
R1
R2
R3
Cumulative sgRNA frequency
Norm. sgRNA counts (Log2)
G
Proliferation effect
FDR (−log10)
Fold change Unsorted/plasmid library (log2)
MAPK
X−linked
X-linked MAPK inhibitor

Figure S1. Identification of X-chromosomal MAPK regulators through a pooled CRISPR knockout screen.

(A) Schematic description of the MAPK signaling pathway. Briefly, the fibroblast growth factor (Fgf) binds and activates the Fgf receptor (FgfR), leading to the formation of a complex consisting of the FgfR Substrate 2 (Frs2) and growth factor receptor-bound protein 2 (Grb2). This complex activates the small GTPase Ras which triggers the kinase cascade of Raf, Mek and Erk. Phosphorylated Erk translocates to the nucleus and activates MAPK target genes. Erk-dependent deactivation of Raf constitutes a strong negative feedback loop.

(B) Flow cytometry measurement of GFP fluorescence in 1.8-SRE-Elk ESCs, treated for 48 h with 1 µM of the Mek inhibitor U0126 (Meki treated) or with DMSO (Untreated). The parental 1.8 mESC line is shown in grey.

(C) sgRNA distribution in the cloned GeCKOx library.

(D) Heatmap showing the pearson correlation coefficients between the sgRNA counts of all unsorted and double-sorted fractions in the MAPK screen.

(E) Mean fold change between the double-sorted and unsorted populations for non-targeting controls and for individual sgRNAs targeting significantly enriched or depleted genes (FDR < 0.05, MAGeCK), * $p < 0.05$, Wilcoxon rank sum test.

(F) sgRNA distribution in sorted and unsorted fractions in all 3 replicates.

(G) To detect genes that affect growth of mESCs, the sgRNA abundance was compared in the unsorted cells (day 7 after transduction) and the cloned plasmid GeCKOx library. Genes with a FDR<0.05 (MAGeCK) are highlighted in red (MAPK controls) and blue (X-linked genes). Triangles mark the identified X-linked MAPK inhibitors (compare Fig. 1C).

Figure S2

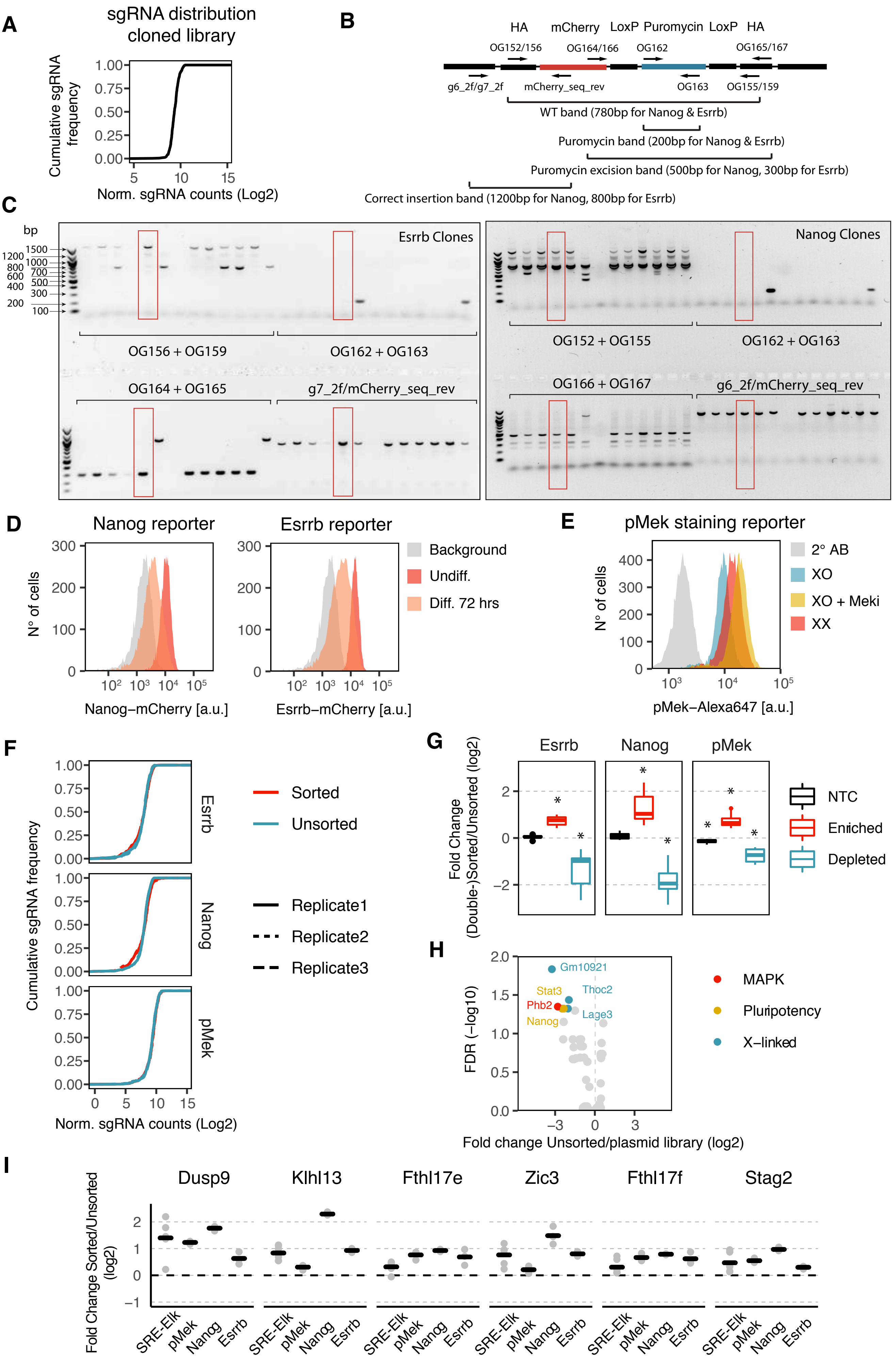

A
sgRNA distribution cloned library
Cumulative sgRNA frequency
Norm. sgRNA counts (Log2)
B
HA
mCherry
LoxP
Puromycin
LoxP
HA
OG152/156
OG164/166
OG162
OG165/167
g6_2f/g7_2f
mCherry_seq_rev
OG163
OG155/159
WT band (780bp for Nanog & Esrrb)
Puromycin band (200bp for Nanog & Esrrb)
Puromycin excision band (500bp for Nanog, 300bp for Esrrb)
Correct insertion band (1200bp for Nanog, 800bp for Esrrb)
C
bp
Esrrb Clones
Nanog Clones
OG156 + OG159
OG162 + OG163
OG164 + OG165
g7_2f/mCherry_seq_rev
OG152 + OG155
OG166 + OG167
g6_2f/mCherry_seq_rev
D
Nanog reporter
Esrrb reporter
N° of cells
Background
Undiff.
Diff. 72 hrs
Nanog−mCherry [a.u.]
Esrrb−mCherry [a.u.]
E
pMek staining reporter
2° AB
XO
XO + Meki
XX
pMek−Alexa647 [a.u.]
F
Cumulative sgRNA frequency
Esrrb
Nanog
pMek
Sorted
Unsorted
Replicate1
Replicate2
Replicate3
Norm. sgRNA counts (Log2)
G
Fold Change (Double-)Sorted/Unsorted (log2)
Esrrb
Nanog
pMek
NTC
Enriched
Depleted
H
FDR (−log10)
Gm10921
Thoc2
Stat3
Phb2
Nanog
Lage3
MAPK
Pluripotency
X−linked
Fold change Unsorted/plasmid library (log2)
I
Fold Change Sorted/Unsorted (log2)
Dusp9
Klhl13
Fthl17e
Zic3
Fthl17f
Stag2
SRE-Elk
pMek
Nanog
Esrrb

Figure S2. Secondary CRISPR screens profiling pluripotency factors, differentiation kinetics and Mek phosphorylation

(A) sgRNA distribution of the cloned GeCKOxs library.

(B-C) Genotyping of Nanog and Esrrb tagged cell lines. (B) Primer locations are shown as arrows and are labelled with the primer number. The expected amplicons are shown below. (C) Genotyping PCR results using the indicated primer pairs, clones used for the secondary screens are highlighted in red.

(D) Flow cytometry measurement of 1.8-Nanog-mCherry (left) and 1.8-Esrrb-mCherry mESCs (right) before and after 3 days of differentiation, showing the expected differentation-induced down-regulation of the two pluripotency factors.

(E) Intracellular pMek staining of 1.8 XX, XO and XO cells treated with Mek inhibitor (Meki) PD0325901 for 48 h, showing the expected feedback-mediated increase in XX mESCs and upon Meki treatment.

(F) Cumulative sgRNA distribution in unsorted and sorted fractions for all replicates of all 3 secondary screens.

(G) Mean fold change between the (double-)sorted and unsorted populations for non-targeting controls and individual sgRNAs targeting significantly enriched or depleted genes (* $p < 0.05$, Wilcoxon rank sum test).

(H) To detect genes that affect growth of mESCs, the sgRNA abundance was compared in the unsorted cells (day 7 after transduction) and the cloned plasmid GeCKOxs library. Genes with a FDR<0.05 (MAGeCK) are highlighted.

(I) Screen results for all individual sgRNAs targeting enriched candidate genes shown in main Fig. 2H.

Figure S3

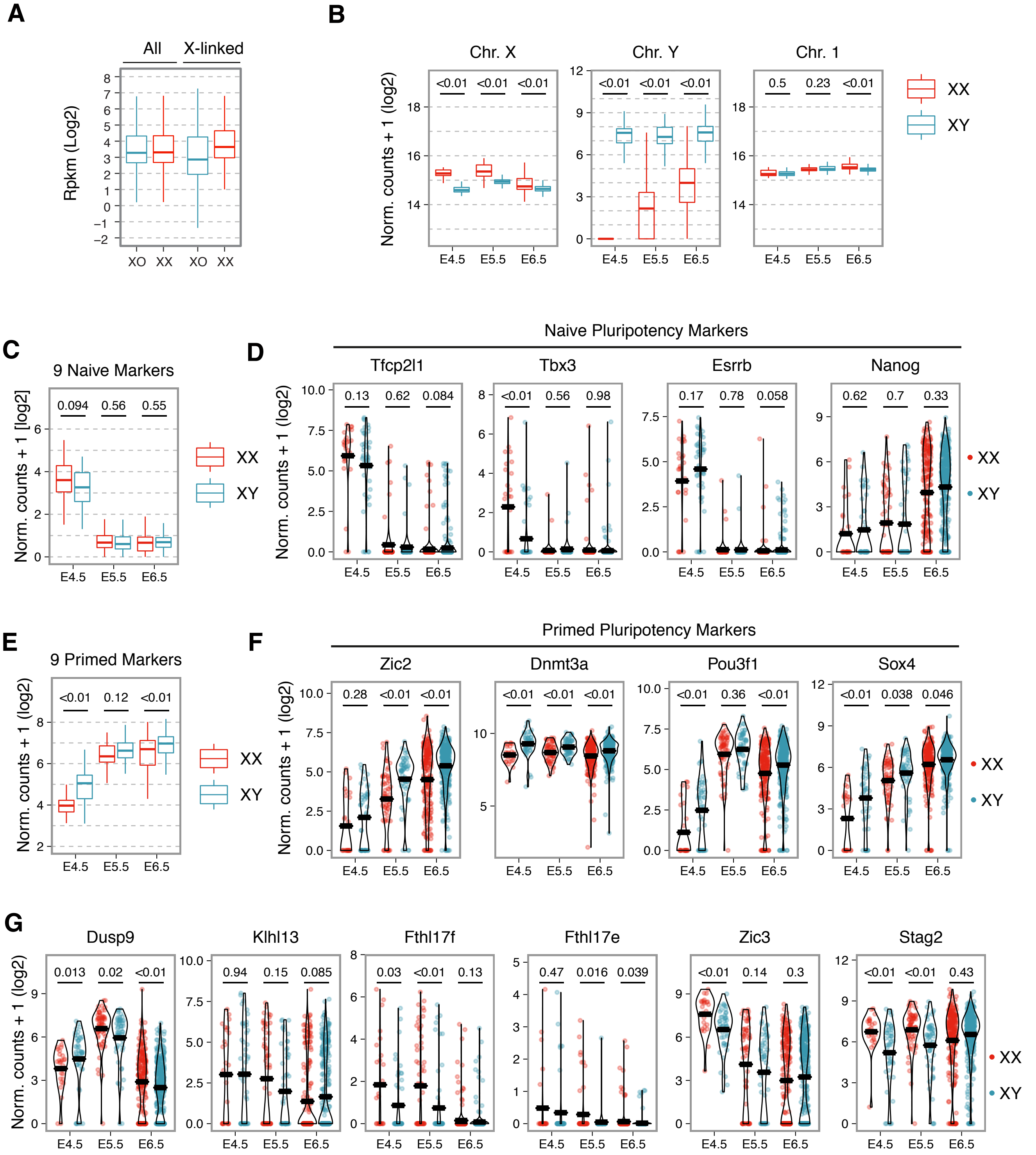

Figure S3. Sex differences in embryonic stem cells and mouse embryos

(A) Boxplot showing expression levels of all genes and only X-linked genes in 1.8 XX and 1.8 XO mESCs.

(B-G) Comparison of male and female epiblast cells at three time points of embryonic development by single-cell RNA sequencing (Argelaguet et al, Nature, 2019). Expression in individual cells are shown for entire chromosomes (B), mean expression for each cell of 9 naive pluripotency factors (Tfcp2l1, Tbx3, Esrrb, Rex1, Klf2, Klf5, Tcl1, Nanog, Klf4) (C), expression of 4 naive pluripotency factors (D), mean expression for each cell of 9 primed pluripotency factors (Fgf5, Otx2, Pou3f1, Dnmt3b, Lef1, Sox4, Zic2, Sall2, Dnmt3a) (E), expression of 4 primed pluripotency factors (F) and expression of X-linked genes identified through CRISPR screening (G). Thick bars in D,F,G indicate the mean. p-values for a Wilcoxon rank sum test are indicated.

Figure S4

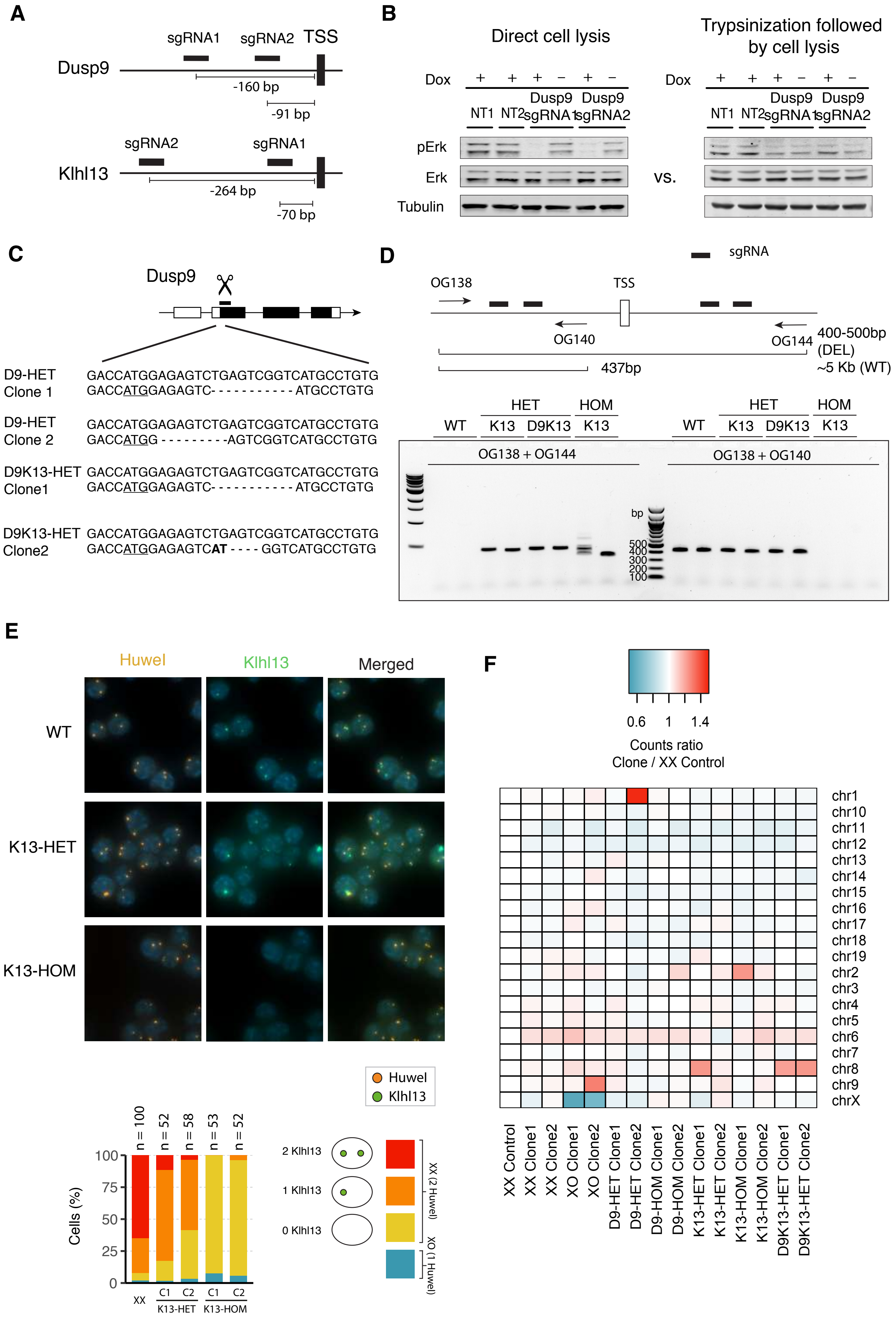

A
sgRNA1
sgRNA2
TSS
Dusp9
-160 bp
-91 bp
sgRNA2
sgRNA1
Klhl13
-264 bp
-70 bp
B
Direct cell lysis
Trypsinization followed by cell lysis
Dox
NT1
NT2
Dusp9 sgRNA1
Dusp9 sgRNA2
pErk
Erk
Tubulin
vs.
C
Dusp9
D9-HET Clone 1
GACCATGGAGAGTCTGAGTCGGTCATGCCTGTG
GACCATGGAGAGTC-----------ATGCCTGTG
D9-HET Clone 2
GACCATGGAGAGTCTGAGTCGGTCATGCCTGTG
GACCATGG---------AGTCGGTCATGCCTGTG
D9K13-HET Clone1
GACCATGGAGAGTCTGAGTCGGTCATGCCTGTG
GACCATGGAGAGTC-----------ATGCCTGTG
D9K13-HET Clone2
GACCATGGAGAGTCTGAGTCGGTCATGCCTGTG
GACCATGGAGAGTCAT----GGTCATGCCTGTG
D
sgRNA
OG138
TSS
OG140
OG144
400-500bp (DEL)
~5 Kb (WT)
437bp
HET
HOM
WT
K13
D9K13
OG138 + OG144
OG138 + OG140
bp
500
400
300
200
100
E
Huwel
Klhl13
Merged
WT
K13-HET
K13-HOM
Huwel
Klhl13
n = 100
n = 52
n = 58
n = 53
n = 52
Cells (%)
100
75
50
25
0
XX
C1
C2
K13-HET
K13-HOM
2 Klhl13
1 Klhl13
0 Klhl13
XX (2 Huwel)
XO (1 Huwel)
F
0.6
1
1.4
Counts ratio
Clone / XX Control
chr1
chr10
chr11
chr12
chr13
chr14
chr15
chr16
chr17
chr18
chr19
chr2
chr3
chr4
chr5
chr6
chr7
chr8
chr9
chrX
XX Control
XX Clone1
XX Clone2
XO Clone1
XO Clone2
D9-HET Clone1
D9-HET Clone2
D9-HOM Clone1
D9-HOM Clone2
K13-HET Clone1
K13-HET Clone2
K13-HOM Clone1
K13-HOM Clone2
D9K13-HET Clone1
D9K13-HET Clone2

Figure S4. Perturbation of Klhl13 and Dusp9 in mESCs

(A) Position of the sgRNA sequences used to over-express Dusp9 and Klhl13 in Fig. 3. SgRNAs for Dusp9 were targeted 91 bp (chrX:73,639,328-73,639,346, GRCm38/mm10 Assembly) and 160 bp (chrX: 73,639,259-73,639,277) upstream of the transcriptional start site (TSS), whereas for Klhl13 they were targeted 264 bp (chrX:23,365,328-23,365,347) and 70 bp (chrX:23,365,134-23,365,152) upstream of the TSS.

(B) Comparison of pErk levels in cells over-expressing Dusp9, harvested either through direct cell lysis (left) or after trypsinization and cell pelleting (right).

(C) Sequence of Dusp9 frameshift mutants, the start codon is underlined.

(D) PCR-genotyping of Klhl13 mutant lines. Arrows indicate primer position, thick bars sgRNA target sites.

(E) RNA fluorescence in situ hybridization of wildtype, heterozygous and homozygous Klhl13 mutant mESCs using probes for Klhl13 (green) and another X-linked gene (Huwe1, orange). The fraction of cells with a specific pattern was quantified (bottom).

(F) Karyotyping of all cell lines used in Fig. 4 by double digest genotyping by sequencing (ddGBS). Counts mapping to each chromosome were normalized to an XX clone that had previously been karyotyped via metaphase spreads.

Figure S5

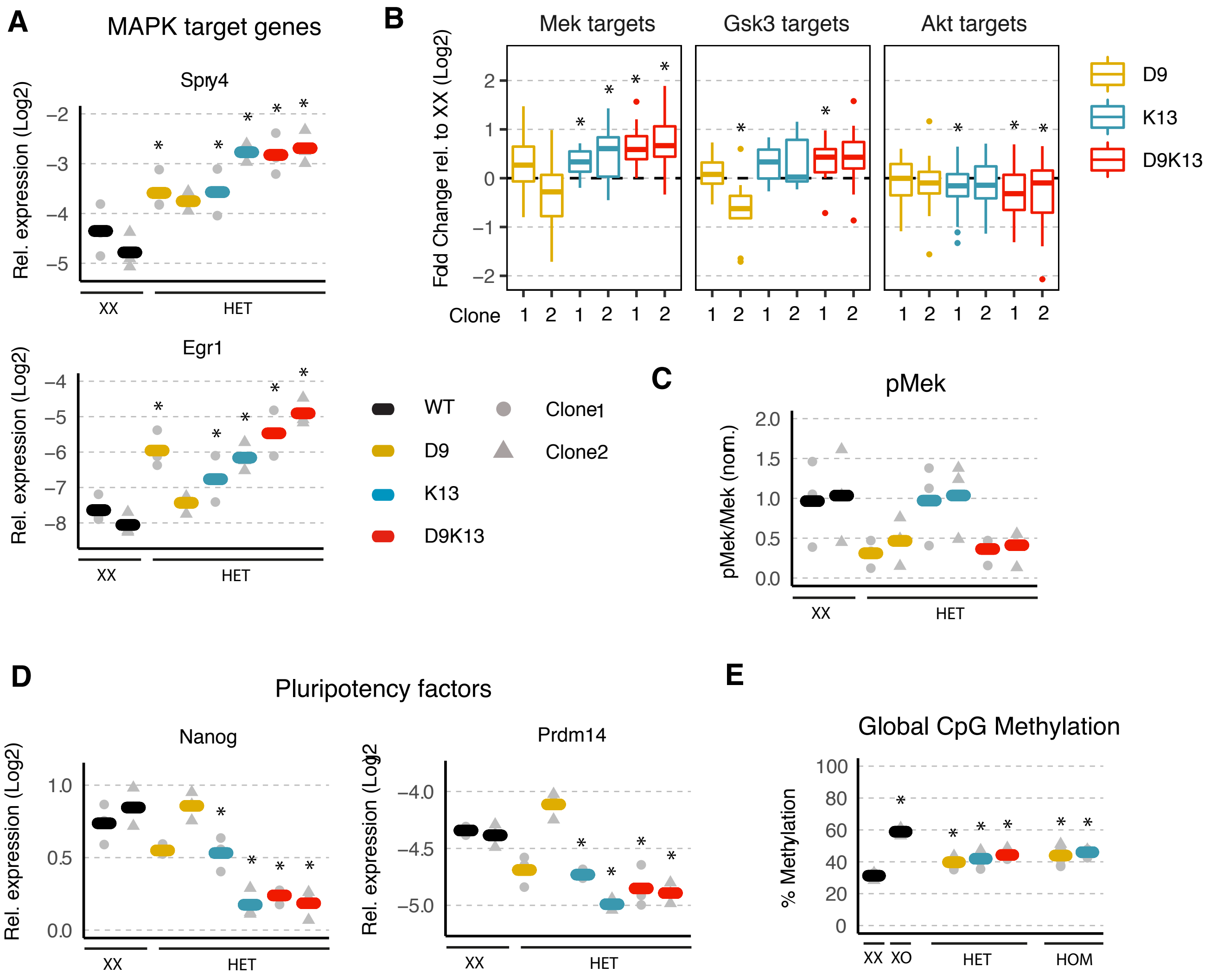

Figure S5. Heterozygous mutations of Klhl13 and Dusp9 in female mESCs partially phenocopy the male pluripotency state

(A-D) Comparison of female 1.8 XX mESCs with a heterozygous (HET) deletion of Dusp9 (yellow), Klhl13 (blue) or both (red) with the parental XX line (2 clones per genotype plotted separately). Individual measurements are shown as grey dots (clone 1) and triangles (clone 2) and the mean across three biological replicates for each clone is indicated by a thick bar. (A) Quantification of MAPK target genes by qPCR. (B) Boxplots showing expression of Mek (left), Gsk3 (middle) and Akt (right) target genes in each clone for cell lines with the indicated genotype, normalized to the average expression in two XX clones, as assessed by RNA-seq. Boxes indicate the 25th to 75th percentiles and the central line represents the median. (C) Quantification of pMek, normalized to total Mek and to the XX control cells by immunoblotting. (D) Pluripotency factor expression (Nanog and Prdm14) assessed by qPCR. * $p < 0.05$ Wilcoxon rank sum test (B), otherwise two-tailed paired Student's t-test comparing each clone for a given mutant cell line and the mean of the two analyzed XX wildtype control clones. (E) Global CpG methylation levels assessed via pyrosequencing-based luminometric DNA methylation assay (LUMA) in the cell lines used in Fig. 4 in Serum/LIF conditions. Individual measurements are shown as grey dots (clone 1) and triangles (clone 2) and the mean across two clones and three biological replicates is indicated by a thick bar. *$p < 0.05$ two-tailed paired Student's t-test comparing each mutant/XO cell line with the XX wildtype cells

Figure S6

A

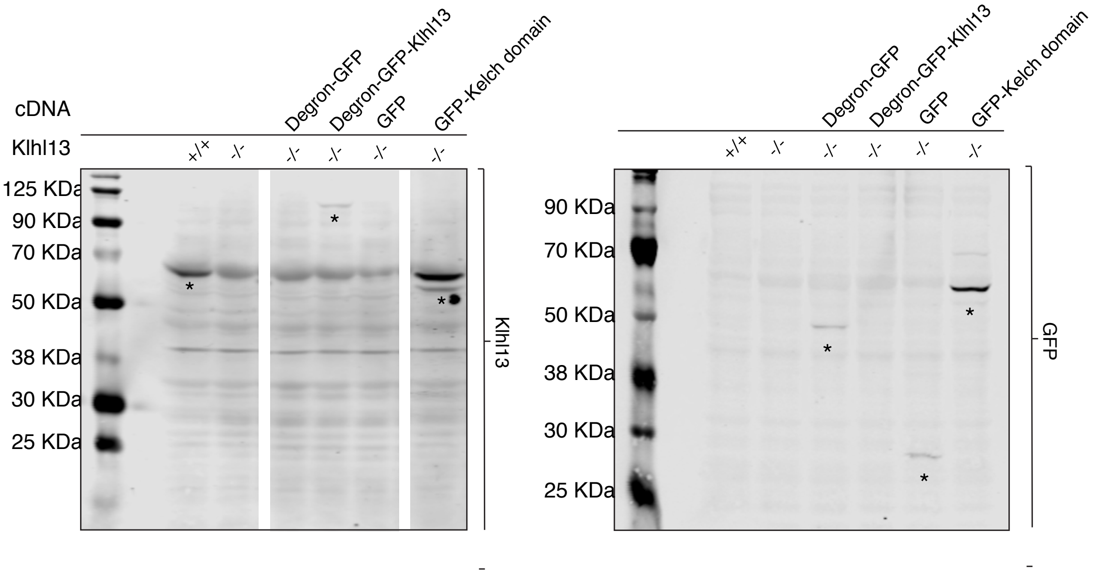

Figure S6. Identification of Klhl13 target proteins that mediate its effect on pluripotency and differentiation

(A) Immunoblotting of protein lysates from female K13-HOM cell lines expressing constructs for the identification of Klhl13 interacting proteins and an XX wildtype control. Membranes were incubated with an anti-Klhl13 antibody (left) and an anti-GFP antibody (right). * bands with the expected size.

Figure S7

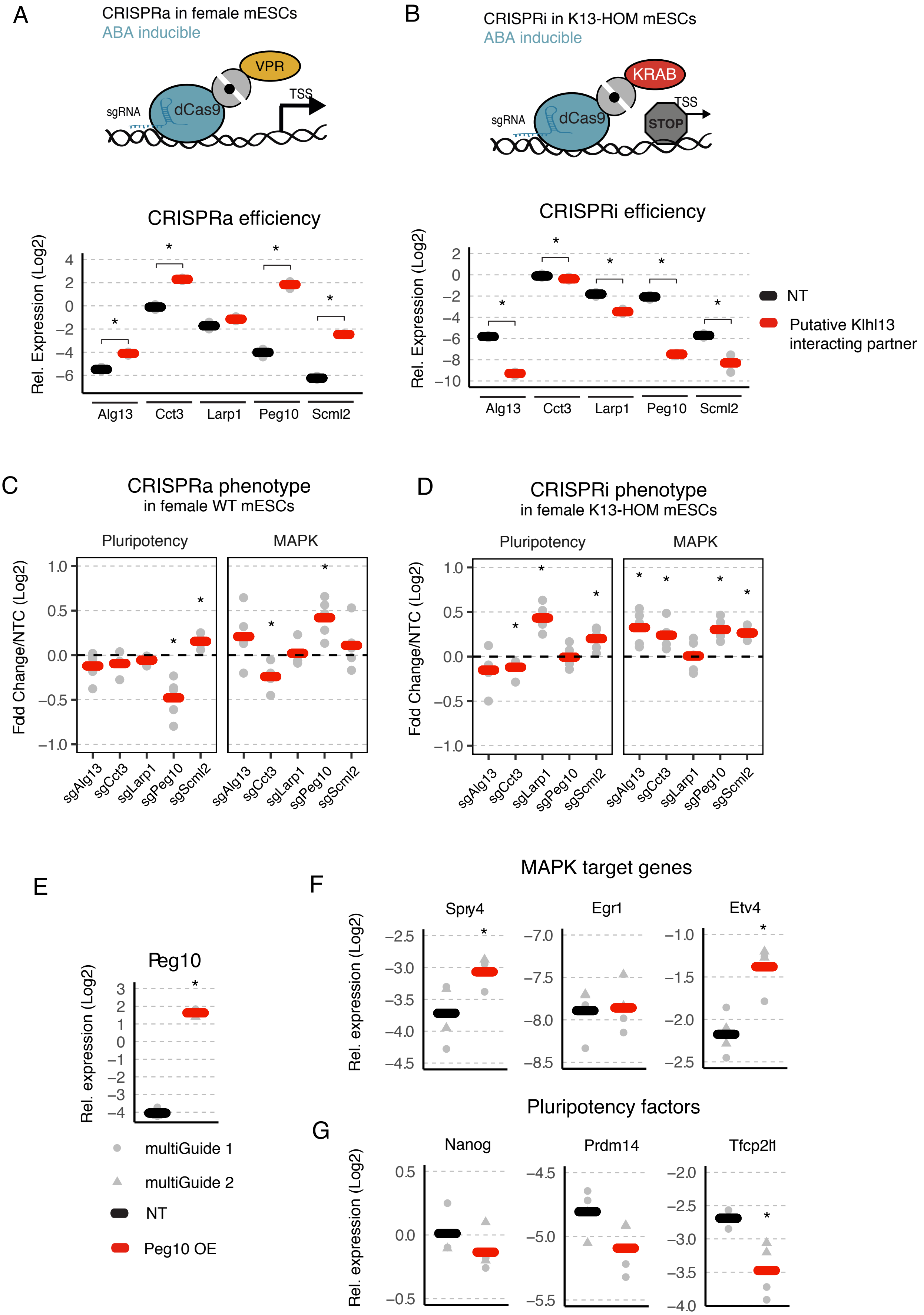

A
CRISPRa in female mESCs
ABA inducible
VPR
sgRNA
dCas9
TSS
B
CRISPRi in K13-HOM mESCs
ABA inducible
KRAB
sgRNA
dCas9
STOP
TSS
CRISPRa efficiency
Rel. Expression (Log2)
Alg13
Cct3
Larp1
Peg10
Scml2
CRISPRi efficiency
Rel. Expression (Log2)
Alg13
Cct3
Larp1
Peg10
Scml2
NT
Putative Klhl13 interacting partner
C
CRISPRa phenotype
in female WT mESCs
Pluripotency
MAPK
Fold Change/NTC (Log2)
sgAlg13
sgCct3
sgLarp1
sgPeg10
sgScml2
D
CRISPRi phenotype
in female K13-HOM mESCs
Pluripotency
MAPK
Fold Change/NTC (Log2)
sgAlg13
sgCct3
sgLarp1
sgPeg10
sgScml2
E
Peg10
Rel. expression (Log2)
multiGuide 1
multiGuide 2
NT
Peg10 OE
F
MAPK target genes
Spry4
Egr1
Etv4
Rel. expression (Log2)
G
Pluripotency factors
Nanog
Prdm14
Tfcp2l1
Rel. expression (Log2)

Figure S7. Effects of putative Klhl13 targets proteins on MAPK target gene and pluripotency factor expression

(A-B) 1.8 female wildtype mESCs stably expressing the CRISPRa system (A) and 1.8 female K13-HOM mESCs stably expressing the indicated CRISPRi system (B) were transduced with vectors expressing sgRNAs targeting the identified putative Klhl13 target proteins Alg13, Cct3, Larp1, Peg10 and Scml2 (red) or NTCs (black). Expression of each gene was quantified by qPCR in cells expressing the respective sgRNAs or NTCs, as indicated. Individual biological replicates (n=3) are depicted as grey dots and the mean is indicated by a thick bar. Cells were treated with abscisic acid (ABA) for five days prior to cell harvesting for phenotypic assessment.

(C-D) Expression levels assessed by qPCR of five MAPK target genes (Spry4, Egr1, Etv4, Dnmt3b, Grhl2) and five pluripotency factors (Nanog, Prdm14, Tfcp2l1, Tbx3 and Tcl1) in the cells shown in (A) for (C) and in (B) for (D). Grey dots represent the mean expression of each of the five genes across three biological replicates for cells expressing the respective sgRNAs normalized to the mean expression for cells expressing NTCs for each gene, and bars represent the mean of the five assessed genes.

(E-G) 1.8 female wildtype mESCs stably expressing the CRISPRa system in (A) were transduced with two different vectors (multiGuide 1 and 2) expressing sgRNAs targeting Peg10 (red) or NTCs (black). Expression levels of Peg10 (E), MAPK target genes (F) and pluripotency factors (G) were quantified by qPCR in cells expressing the two different Peg10 targeting sgRNA or NTC vectors, as indicated. Individual measurements are shown as grey dots (multiGuide construct 1) and triangles (multiGuide construct 2) and the mean across the two multiGuide constructs and two biological replicates is indicated by a thick bar. Cells were treated with abscisic acid (ABA) for five days prior to cell harvesting for phenotypic assessment. * $p < 0.05$ one-sample Student's t-test (D-E), otherwise two-tailed paired Student's t-test comparing gene-specific sgRNAs and NTCs are indicated.
