# Supplementary material for: Identification of X-chromosomal genes that drive sex differences in embryonic stem cells through a hierarchical CRISPR screening approach
Source: Genome Biol. 2021 Apr 16;22:110. doi: 10.1186/s13059-021-02321-2 (PMC8051100; doi:10.1186/s13059-021-02321-2)
Supplement: Supplementary file 10 — Additional file 10. Review history. [file 13059_2021_2321_MOESM10_ESM.docx]

Review History

**First round of review**

**Reviewer 1**

**Are you able to assess all statistics in the manuscript, including the appropriateness of statistical tests used?**

No, I do not feel adequately qualified to assess the statistics.

**Comments to author:**

The article GBIO-D-21-00215 "Identification of X-chromosomal genes that drive sex differences in embryonic stem cells through a hierarchical CRISPR screening approach" by Genolet et al. describes a number of approaches to discover mediators of sex-specific differences in ESCs. For this, the authors employ a series of genetic screens for X-Chromosomal factors influencing MAPK signalling and pluripotency and a number of additional strategies (CRISPRa, CRISPRi, IP-MS) to characterize two hits Klhl13 and Dusp9, further. Although the outcome of each individual experiment would already be worth reporting, the article profits from a large amount of data collected from well conducted and described experiments. This breadth and quality of experiments calls for a publication without extensive revision, despite the fact that Dusp9 is not anymore novel in the scientific context and many interesting follow-up experiments could of course be suggested.

Smaller points:

-	The authors choose not only to analyse ESCs mutated for Klhl13 and Dusp9 individually, but also in combination. Unfortunately, in the CRISPRa approach they omit the multiplexed approach, although it should be possible to activate both (and potentially many other screening hits) in parallel, since gRNA vectors with multiple cassettes have been reported.

-	Similarly, it would be interesting to expand the transcriptomic analysis to the CRISPRa approach and characterize the results from the mutant lines (Figure 5) further (What kind of genes, GO-families etc. are common and different in the Venn diagram?).

-	It seems it would be beneficial for the structure of the article if the characterization of the Klhl13 interactors would be described earlier in the text and the functional analysis of their manipulation with (or directly after) that of Klhl13 and Dusp9.

**Reviewer 2**

**Are you able to assess all statistics in the manuscript, including the appropriateness of statistical tests used?**

No, I do not feel adequately qualified to assess the statistics.

**Comments to author:**

The question why female mouse ESCs with two active X chromosomes have a different transcriptional (pluripotency), signaling and DNA-methylation state compared to male ESCs with one active X chromosome has been an important question in the stem cell field and for understanding mechanistically how two active X chromosomes can alter cell states. It is thought that the double dose of X-linked genes in female naïve pluripotent cells slows down female development slightly, enabling robust X-chromosome inactivation and possibly other sex-specific processes. Consequently a huge interest exists to define the X-linked genes that lead to the state differences between female and male ESCs. In the past, Dusp9 has been shown to underlie sex differences in DNA methylation, yet, pluripotency-related gene expression was not affected by the heterozygous Dusp9 deletion. Thus, additional X-linked genes are likely controlling these processes. The authors address this problem systematically by performing several well thought of pooled Crispr screens. The first screen assayed which X-linked regulators control MAPK signaling. In a second set of screens, regulators of proliferation were ruled out and the top candidates from the first screen were pushed into screens for pluripotency factor expression, differentiation dynamics and phosphorylation of Mek, isolating cells with a male ESC-like expression and phosphorylation pattern. I find this approach very good as it was unbiased and did not make conclusions solely based on the proposed function of the respective X-linked genes. Thus, this is an exciting approach to study how X chromosome dosage controls cell state. These screens identified Klhl13 and Dusp9 as key regulators (also based on the demonstration that these genes are indeed more highly expressed in female versus male ESCs and post-implantation embryos). Although Dusp9 had been implicated before (based on its role in erk signaling), Klhl13 was not known as regulator of the female ESC-specific state. The authors then generated a lot of different cell lines to over express DUSP9 or Klhl13 in male ESCs (looking for a female phenotype) and deleting or depleting them alone or together in female ESCs (looking for a male specific phenotype). Again, they assess MAPK pathway activity, pluripotency expression and also DNA methylation levels (which are normally much lower in female than male ESCs). Interestingly, the overexpression experiments yielded different results for Dusp9 and Klhl13, yielding a shift to the female pluripotency program and low DNA methylation for Dusp9 only. Conversely, the heterozygous deletion of Klhl13 in female cells has a stronger effect on pluripotency factor expression and differentiation than Dusp9, and

the double mutant female ESCs become much more male ESC like (although not completely). Thus it seems that Dusp9 is responsible for the reduced levels of MAPKinase phosphorylation and low DNA methylation, and that the pluripotency and differentiation phenotypes signatures can be largely attributed Klhl13. The authors end up with identifying interaction partners of Klhl13, yet none of them alone seems to mediate the effects of Klhl13. This likely the downstream pathways are complex. Overall, the paper shows that Dusp9 and Klhl13 control different aspects of the X-dosage induced phenotype and that they appear to act in concert with additional factors. I don't have any major concerns about this manuscript and think it is important to publish both the approach and findings.

Minor comments:

-	Please clarify if Figure 5 is only showing data for autosomal genes.

-	It would be nice to see the expression level of the downstream targets of Klhl13 in female and male ESCs/embryos.

**Authors Response**

**Point-by-point responses to the reviewers’ comments:**

Reviewer #1:

Smaller points:

Reviewer: The authors choose not only to analyse ESCs mutated for Klhl13 and Dusp9 individually, but also in combination. Unfortunately, in the CRISPRa approach they omit the multiplexed approach, although it should be possible to activate both (and potentially many other screening hits) in parallel, since gRNA vectors with multiple cassettes have been reported.

*Answer: While we agree that the suggested experiments would be worthwhile, we think they are beyond the scope of this work.*

Reviewer: Similarly, it would be interesting to expand the transcriptomic analysis to the CRISPRa approach and characterize the results from the mutant lines (Figure 5) further (What kind of genes, GO-families etc. are common and different in the Venn diagram?).

*Answer: A transcriptomic analysis of the CRISPRa experiment would without doubt be interesting. However, we feel this is not required to support the conclusions of our manuscript.*

Reviewer: It seems it would be beneficial for the structure of the article if the characterization of the Klhl13 interactors would be described earlier in the text and the functional analysis of their manipulation with (or directly after) that of Klhl13 and Dusp9.

*Answer: We have seriously considered to restructure the manuscript as suggested. However, one central component of our strategy to identify putative Klhl13 target proteins (Fig. 7) is the identification of proteins that are more abundant in homozygous Klhl13 knock-out cells compared to the wildtype. Since we describe generation and characterization of this mutant cell line only in Fig. 4 as part of the functional analysis of the screen hits, it would be difficult to describe the identification of Klhl13 target proteins before the functional validation in Fig. 3-6. We would therefore prefer to keep the current structure of the manuscript.*

Reviewer #2:

Minor comments:

Reviewer: Please clarify if Figure 5 is only showing data for autosomal genes.

*Anwer: The differential expression analysis and the PCA plot in Fig. 5 were indeed generated with autosomal genes only. In the revised version of the manuscript we state this explicitly in the figure legend, in the text and in the methods section.*

Reviewer: It would be nice to see the expression level of the downstream targets of Klhl13 in female and male ESCs/embryos.

*Answer: Here we are not entirely sure what the reviewer means. If “downstream targets of Klhl13” refers to the putative target proteins we identified, we would actually not expect to see any sex differences on the transcriptomic level, which is assayed in the embryo and mESC data. This analysis would be very interesting on the protein level, where we would expect increased protein abundance in male cells. Unfortunately, this data is not available.*
